# Age-Dependent Increase of Absence Seizures and Intrinsic Frequency Dynamics of Sleep Spindles in Rats

**DOI:** 10.1155/2014/370764

**Published:** 2014-06-23

**Authors:** Evgenia Sitnikova, Alexander E. Hramov, Vadim Grubov, Alexey A. Koronovsky

**Affiliations:** ^1^Institute of the Higher Nervous Activity and Neurophysiology of Russian Academy of Sciences, Butlerova Street 5A, Moscow 117485, Russia; ^2^Research and Educational Center “Nonlinear Dynamics of Complex Systems”, Saratov State Technical University, Saratov, Polytechnicheskaya Street 77, Saratov 410054, Russia; ^3^Faculty of Nonlinear Processes, Saratov State University, Saratov, Astrakhanskaya Street 83, Saratov 410012, Russia; ^4^Saratov State University, Astrakhanskaya Street 83, Saratov 410012, Russia

## Abstract

The risk of neurological diseases increases with age. In WAG/Rij rat model of absence epilepsy, the incidence of epileptic spike-wave discharges is known to be elevated with age. Considering close relationship between epileptic spike-wave discharges and physiologic sleep spindles, it was assumed that age-dependent increase of epileptic activity may affect time-frequency characteristics of sleep spindles. In order to examine this hypothesis, electroencephalograms (EEG) were recorded in WAG/Rij rats successively at the ages 5, 7, and 9 months. Spike-wave discharges and sleep spindles were detected in frontal EEG channel. Sleep spindles were identified automatically using wavelet-based algorithm. Instantaneous (localized in time) frequency of sleep spindles was determined using continuous wavelet transform of EEG signal, and intraspindle frequency dynamics were further examined. It was found that in 5-months-old rats epileptic activity has not fully developed (preclinical stage) and sleep spindles demonstrated an increase of instantaneous frequency from beginning to the end. At the age of 7 and 9 months, when animals developed matured and longer epileptic discharges (symptomatic stage), their sleep spindles did not display changes of intrinsic frequency. The present data suggest that age-dependent increase of epileptic activity in WAG/Rij rats affects intrinsic dynamics of sleep spindle frequency.

## 1. Introduction

Sleep spindles are well-known EEG phenomena that reflect spontaneous rhythmic activity of thalamocortical neuronal network during non-REM sleep [1–3]. In vivo experiments demonstrated a close relationship between sleep spindles and epileptic spike-wave discharges (SWD) [4–6]. SWD are electroencephalographic (EEG) manifestation of absence epilepsy, and they are triggered by the cortex, opposite to sleep spindles, which are known to originate from the thalamus (reviewed in [7]). In comparison to sleep spindles, SWD are underlain by more intensive excitation and/or synchronization processes in thalamocortical network [7–9]. Previously we demonstrated that sleep spindles and SWD showed different time-frequency characteristics, as measured in the cortex and thalamus [10].

Intraspindle frequency is an important parameter characterizing intrinsic properties of thalamocortical network activity [11, 12] with respect to generation of autonomous oscillations. In healthy human subjects, the frequency of sleep spindles is known to vary from 10 to 16 Hz (e.g., [2, 12, 13]), and in animals (rats and cats) from 7 to 14 Hz [1, 14]. Recently we compared time-frequency characteristics of the anterior sleep spindles in nonepileptic Wistar and epileptic WAG/Rij rats (genetic model of absence epilepsy) [15] and demonstrated that instantaneous frequency of sleep spindles in symptomatic WAG/Rij rats was constant during a spindle event, opposite to ascending dynamics of intraspindle frequency in control Wistar rats. We also found [16] that ~50% of anterior sleep spindles in WAG/Rij rats (at the age between 5 and 9 months) appeared in EEG with the frequency between 8 and 10 Hz (mean ~9.3 Hz), 20–25% of spindles—with frequency between 10 and 12 Hz (~11.4 Hz), and 25–30%—between 12 and 14 Hz (~13.4 Hz), whereas an increase of intrinsic frequency during sleep spindle was found at the younger age (5 months) and only in ~9.3 and ~11.4 Hz sleep spindles, but not in ~13.4 Hz spindles. It is well known that the number and duration of SWD in WAG/Rij rats increase with age [17–19], although age-dependent changes in sleep spindles are still uncertain. Considering close relationship between epileptic spike-wave discharges and physiologic sleep spindles, it was assumed that age-dependent increase of epileptic activity may affect time-frequency characteristics of sleep spindles. In order to examine this hypothesis in the current study, we compared dynamics of intraspindle frequency in WAG/Rij rats at the younger age (preclinical state) and elder (symptomatic) ages, when SWD are fully developed in EEG.

## 2. Materials and Methods

Experiments were conducted in six male WAG/Rij rats at the Institute of Higher Nervous Activity and Neurophysiology RAS and were approved by the Ethical Committee on Animal Experimentation of this Institution. EEGs were recorded during three successive sessions at the age of ~5 months (from 4.8 to 5), ~7 months (6.8–7.1), and ~9 months (8.5–9). Rats were equipped with metal screw electrodes that were implanted epidurally at the right hemisphere over the frontal cortex (coordinates: AP +2 mm and L 2.5 mm relative to bregma), parietal (AP −2 mm; L 5.5 mm) and occipital areas (AP −5 mm; L 3 mm) under chloral hydrate anesthesia (325 mg/kg, 4% solution in 0.9% NaCl). Recordings were made continuously during a period of 24 hours in freely moving rats. EEG signals were band-pass filtered between 0.5 and 200 Hz, digitized with 400 samples/second/per channel, and stored on hard disk. Only frontal EEG data were used for time-frequency analysis (because sleep spindles showed maximum amplitude in the frontal channel), while occipital and parietal EEGs were used to facilitate determining the state of vigilance, in particular, slow wave sleep.

**Figure 1:**
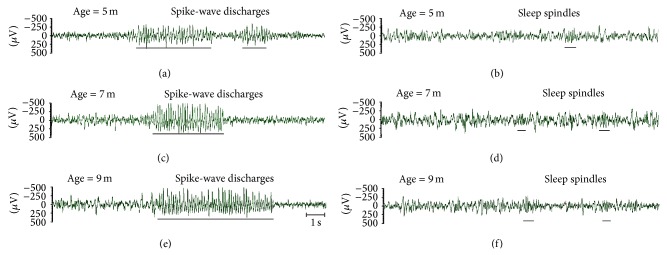
Examples of epileptic activity (spike-wave discharges, SWD) and sleep spindles as recorded in frontal EEG in WAG/Rij rat at different ages. Note that at the age of 5 months, waveform of SWD was immature (waxing-winning envelope, unstable amplitude of spikes in a train).

Sleep spindles and SWD were investigated in frontal EEG (Figure 1) for the reason that they both displayed amplitude maximum in this (anterior) area [5, 8, 14, 17, 19]. SWD were detected visually as a sequence of repetitive high-voltage negative spikes and negative waves that lasted longer than 1 sec; amplitude of SWD exceeded background more than three times [5, 17, 18]. The number and duration of SWD were scored in 6-hour interval during dark phase. Sleep spindles were recognized in EEG as 8–14 Hz waves with characteristic waxing-waning morphology and symmetrical waveform, whose amplitude exceeded background level at least twice.

The continuous wavelet transform (CWT) was used for time-frequency analysis of sleep spindles in EEG as recorded during dark phase. Three to 5 intervals during slow-wave sleep (duration 15–30 s) per animal in each age group were extracted. The CWT, *W*(*s*, *τ*), was obtained by convolving the EEG signal, *x*(*t*), with the basis function *ϕ*_0_

(1) W(s,τ)=1s∫−∞+∞x(t)ϕ0∗(t−τs)dt  “∗”  denotes  complex  conjugation,

where *s*: time scales (that were converted into Fourier frequencies *f*) and *τ*: the time shift.

Complex Morlet wavelet, *ϕ*_0_, was used as basis function

(2) $$\begin{array}{c} \phi_{0}=\frac{1}{\sqrt[{4}]{\pi }}e^{j\Omega \eta }e^{-\eta^{2}/2}, \end{array}$$

in which parameter Ω = 2*π*.

Sleep spindles were detected automatically using earlier developed wavelet-based algorithm in frontal EEG in 15–30 s intervals during slow-wave sleep [15, 16, 20]. Briefly, wavelet energy *w*(*t*) was measured in the spindle frequency band, *F* ∈ (8, 16) Hz

(3) $$\begin{array}{c} w\left(t\right)=\overset{}{\underset{F}{\int }}{\left|W\left(f_{s},t\right)\right|}^{2}df_{s}. \end{array}$$

The value of *w*(*t*) was averaged in the time window *T*:

(4) $$\begin{array}{c} \langle w\left(t\right)\rangle =\overset{t+T}{\underset{t-T}{\int }}w\left(t^{\prime }\right)dt^{\prime }. \end{array}$$

The best quality of automatic recognition was achieved when *T* was set to 0.5 s that roughly corresponded to the averaged duration of a sleep spindle. Finally, the threshold level of wavelet power, *w*_*c*_, was empirically defined and sleep spindles were identified under condition 〈*w*(*t*)〉 > *w*_*c*_. In order to determine the end point of sleep spindles, wavelet power of background EEG was averaged in the frequency band *F* ∈ (8, 16) Hz over the time period of 10 s, *w*_0_. The value of *w*_0_ was compared with the averaged wavelet power in the same band *F* ∈ (8, 16) Hz, 〈*w*(*t*)〉. The end point of sleep spindle was assigned when 〈*w*(*t*)〉 < 0.25*w*_0_. The true positive detections of sleep spindles reached 90–95% of visually selected sleep spindles.

Rapid changes of the dominant frequency during sleep spindles were explored using “skeletons” of wavelet surfaces that were constructed based on the previously described procedure [15, 16]. First, the instantaneous wavelet energy distribution *E*_*i*_(*f*_*s*_, *t*_0_) = |*W*(*f*_*s*_,*t*_0_)|^2^ was computed for the time moment *t*_0_. Second, the function *E*_*i*_(*f*_*s*_, *t*_0_) was examined for the presence of local maxima, *E*_max⁡,*k*_. If several maxima were detected in *E*_*i*_(*f*_*s*_, *t*_0_), the highest maximum was selected and its frequency was considered as the dominant frequency at the given time moment *t*_0_. The value of the dominant frequency was used as the initial point in “skeleton.” In order to plot the full “skeleton” of wavelet surface, the abovementioned procedure was repeated for the next time point. Skeletons of wavelet surfaces were constructed in 5 s time intervals containing sleep spindles.

Nonparametric Friedman's ANOVA was used for the statistical analysis of age-dependent changes of measurable parameters in EEG (with 3 levels of “age,” within-subject design, repeated measures) and Wilcoxon matched pairs test for the subsequent* post hoc* analysis.

## 3. Results

Epileptic activity in WAG/Rij rats increased with age. The number of SWD increased in a period between 5 and 9 months of age from 3 to 38 discharges as counted in 6-hour interval (Friedman test, *χ*_*N*=6, *df*=2_^2^ = 6.5, *P* < 0.05), as well as the total duration of seizure activity as summed in 6 hours (from 34 ± 20 s to 439 ± 281 s, *χ*_*N*=6, *df*=2_^2^ = 8.0, *P* < 0.05). At the age of 5 months, SWD were completely absent in 4 out of 6 rats, and the rest two animals showed very few discharges with immature waveform (waxing-winning envelope, low frequency, and unstable amplitude of spikes, Figure 1). This implies, first, that spike-wave activity in Moscow's population of WAG/Rij rats appeared at the older age as compared to relatively early development of SWD in native population in Nijmegen, in which SWD were known to be fully developed at the age of 5 months [18]. Second, five months of age in our rats might be considered as a preclinical state of absence epilepsy.

In total, 115 sleep spindles were automatically selected in EEG and analyzed in 5-month-old WAG/Rij rats, 117 spindles—in 7-month-old, and 115 spindles—in 9-month-old. Intrinsic frequency dynamics of sleep spindles were examined using “skeletons” of wavelet surfaces constructed in the spindle frequency band, 9–14 Hz. It was found that instantaneous frequency of sleep spindles slightly changed from the beginning to the end of each sleep spindle event.

**Figure 2:**
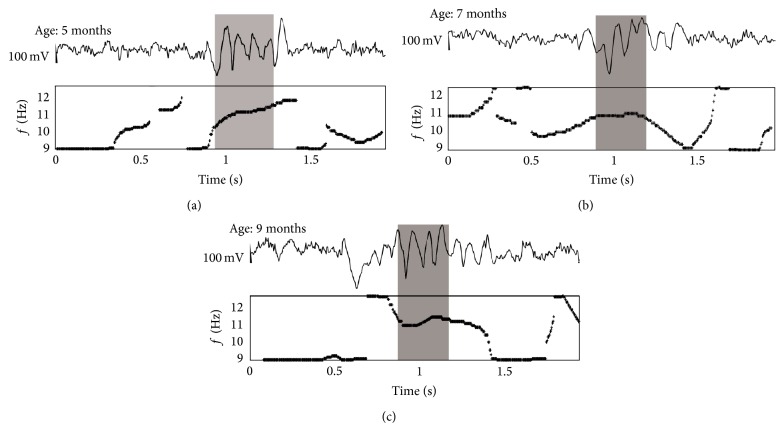
Two-second EEG epochs with sleep spindles as recorded in WAG/Rij rat at three different ages. Sleep spindles (marked in grey) were identified automatically based on the continuous wavelet transform. Bottom plots demonstrate “skeletons” of wavelet surfaces, in which dominant frequency fluctuates in the spindle frequency band, 9–14 Hz.

Figure 2 demonstrates three examples of sleep spindles as recorded in one individual at different ages and corresponding wavelet “skeletons.” An increase of dominant frequency was observed from beginning to the end of a sleep spindle only at the age of 5 months (ascending frequency dynamics in the “skeleton” plot in Figure 2(a)), but it was no longer present at the elder ages (Figures 2(b) and 2(c)).

For the statistical analysis, intraspindle frequency was examined in wavelet “skeletons” by measuring the instantaneous frequency at the beginning (*f*_start_) and at the end (*f*_end_) of each sleep spindle.

**Figure 3:**
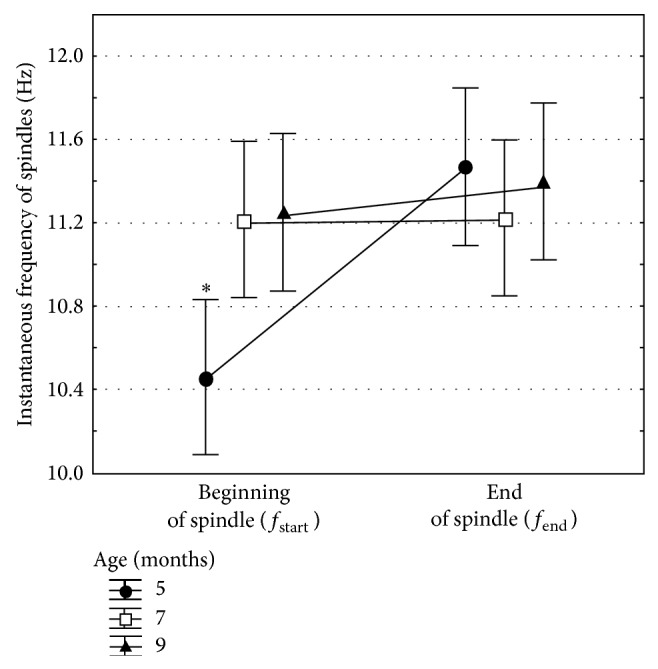
Age-dependent changes of the instantaneous frequency of sleep spindles in WAG/Rij rats as measured at the beginning of a spindle, *f*_start_, and at the end, *f*_end_. Asterisk indicates that instantaneous frequency in 5-month-old WAG/Rij rats was lower than in older ages, and the effect *f*_start_ < *f*_end_ was significant only in 5-month-old WAG/Rij rats (pairwise Wilcoxon tests, all *P*'s <0.05).

It was found, first, that the value of *f*_start_ increased with age (*χ*_*N*=115, *df*=2_^2^ = 12.6,  *P* < 0.005). Second, the value of *f*_start_ at the age of 5 months was lower than that at the age of 7 and 9 months (pairwise Wilcoxon test, all *P*'s <0.05, Figure 3). Third, the difference between *f*_start_ and *f*_end_ significantly changed with age (*χ*_*N*=115, *df*=2_^2^ = 11.3,  *P* < 0.005). According to pairwise Wilcoxon test, ascending intraspindle frequency dynamics, that is, *f*_start_ < *f*_end_, were significant only in 5-month-old WAG/Rij rats (*P* < 0.005), and at the age of 7 and 9 months, the difference between *f*_start_ and *f*_end_ disappeared (*f*_start_ = *f*_end_, Figure 3).

## 4. Discussion

The current paper demonstrates that intrinsic frequency of sleep spindles in WAG/Rij rat model of absence epilepsy changed in parallel to the age-dependent increase of epileptic spike-wave discharges in EEG. Five-month-old WAG/Rij rats developed very few SWD with immature waveform; therefore this age has been considered as preclinical stage. At this age, intrinsic frequency of sleep spindles increased from the beginning to the end. Similar elevation of intraspindle frequency was found previously in nonepileptic Wistar rats at the age of 7 and 9 months [15]. Therefore, in the present study, intraspindle frequency dynamics in 5-month-old WAG/Rij rats were similar to what was previously found in nonepileptic Wistar [15].

Here it was found that the beginning value of spindle frequency (*f*_start_) in preclinical (5 months old) WAG/Rij rats was significantly lower as compared to that in older ages (7 and 9 months), when epileptic discharges became more numerous and epileptic activity became longer. Age-dependent increase of absence seizures in WAG/Rij rats is well known from the literature [17–19, 21]. Considering the present findings, we can add that age-dependent increase of absence seizures was associated with changes of intraspindle frequency, and this might be caused by aggravation of epileptic activity in thalamocortical neuronal network due to more intensive excitation (hyperexcitation) and/or stronger synchronization (hypersynchronization) [7–9]. Furthermore, the low start value of the intraspindle frequency *f*_start_ in preclinical (5 months old) WAG/Rij rats might reflect “normal” rhythmic activity of thalamocortical network.

The current results might shed some light on the controversial data about interrelation between sleep spindles and SWD [7, 8]. According to the present findings, time-frequency profile of sleep spindles in presymptomatic WAG/Rij rats was rather normal (and it was similar to age-matched Wistar rats [15]), but it changed in older ages, when thalamocortical network started producing epileptic discharges. It can be concluded that qualitative changes of sleep spindles were associated with quantitative changes in SWD. It is not surprising, because it is well known that SWD and sleep spindles share the same thalamocortical pathways (reviewed in [1, 7]). Our previous study [21] and the literature [22] indicated that time-frequency properties of SWD changed with age; more specifically, the frequency of immature SWD in younger rats was 5-6 Hz and it increased to 9-10 Hz in older animals. Taken together, both kinds of thalamocortical oscillations, that is, sleep spindles and SWD, displayed age-related changes of time-frequency properties.

In order to explain an increase of intraspindle frequency during sleep spindles in presymptomatic animals, we suggest the following mechanism. It is well known that sleep spindles result from mutual interactions between glutamatergic thalamocortical (TC) neurons in specific thalamic nuclei and GABA-ergic cells in the reticular thalamic nucleus, RTN (refs in [3]). Neurons in RTN have a propensity to trigger spindle oscillations and fire in bursts at every cycle, acting as pacemaker cells. TC cells receive inhibitory synaptic input from the RTN and produce rhythmic bursts only once in two to four cycles. Neuronal network mechanism of sleep spindles includes four processes [1, 23]: (1) initial period—spindle sequence is initiated by the pacemaker cells in the RTN; (2) beginning of a spindle—some TC neurons are silent during the first two-to-four bursts of RTN neurons and do not return signals to the RTN; (3) the middle part of a spindle—all TC cells burst synchronously with RTN neurons; (4) termination of a spindle, which is putatively triggered by the corticothalamic neurons. Here in presymptomatic animals we found that the frequency at beginning of a spindle (the stage 2 of the abovementioned process) was lower than at the middle and at the end (the stage 3). It seems likely that at the stage 3, more TC cells are recruited by RTN, and this results in strengthening of rebound inhibition. Therefore duration of each oscillatory cycle becomes shorter, and the frequency of spindle oscillations increased at the end. In symptomatic animals, the frequency of spindles at beginning (the stage 2) was higher than in presymptomatic animals; therefore, all TC neurons in symptomatic rats might be recruited by the RTN already at the beginning of sleep spindles (similar to the stage 3), and this might lead to the “flattening” of intraspindle frequency. This putative mechanism ought to be investigated in the future. In general, our data indicate that age-dependent development of absence seizures was associated with “flattening” of intrinsic frequency of sleep spindles. The present findings may be helpful for a better understanding of the pathophysiology of absence epilepsy and its probable correlation with sleep disorders, particularly with those related to age.

Specific dynamics of intraspindle frequency were also described in human sleep EEG. Changes of intraspindle frequency (spectral “chirp”) in humans were investigated with the aid of Matching Pursuit algorithm [23, 24]. In particular, Schönwald et al. [24] studied sleep spindles in C3-A2 EEG channel in healthy subjects and showed the higher proportion of negatively chirping spindles, suggesting that sleep spindles in healthy humans tend to decelerate their frequency before termination. In subjects with moderate obstructive sleep apnea, there was a decrease in negatively chirping sleep, or loss of sleep spindle deceleration. This effect was found only in slow spindles and only in frontal and parietal regions [25]. In the present study, flattening of intraspindle frequency dynamics from *f*_start_ < *f*_end_ in preclinical WAG/Rij rats to *f*_start_ = *f*_end_ in symptomatic animals might be considered as negative sign that prerequisites development of epileptic spike-wave discharges.

We believe that our findings would benefit development of new methods for the early (preclinical) diagnosis of epileptic diseases based on time-frequency properties of EEG. Developing of new strategies acting at preclinical stage (preventing epilepsy in a high-risk group) may be considered as future directions in research.

## 5. Conclusions

Continuous wavelet transform was used for time-frequency EEG analysis of sleep spindles in rats with genetic predisposition to absence epilepsy (WAG/Rij). It was found that younger subjects at preclinical stage (5 months old) displayed elevation of intraspindle frequency from the beginning to the end of sleep spindles, but older subjects with fully blown seizures (at the age of 7 and 9 months, symptomatic stage) did not display any changes of intraspindle frequency. This assumes that age-dependent elevation of epileptic activity in WAG/Rij rats affects intrinsic dynamics of sleep spindle frequency.

## Abbreviations

CWT: Continuous wavelet transform
SWD: Spike-wave discharges
TC: Thalamocortical neurons
RTN: The reticular thalamic nucleus.
